# Whole-Genome Sequences of Influenza A(H1N1)pdm09 Virus Isolates from Kerala, India

**DOI:** 10.1128/genomeA.00598-17

**Published:** 2017-07-13

**Authors:** Sara Jones, Raji Prasad, Anjana S. Nair, Sanjai Dharmaseelan, Remya Usha, Radhakrishnan R. Nair, Radhakrishna Madhavan Pillai

**Affiliations:** a Viral Disease Biology Program, Rajiv Gandhi Centre for Biotechnology, Thiruvananthapuram, Kerala, India; b Microbiome Biology Program, Rajiv Gandhi Centre for Biotechnology, Thiruvananthapuram, Kerala, India; c Laboratory Medicine and Molecular Diagnostics Program, Rajiv Gandhi Centre for Biotechnology, Thiruvananthapuram, Kerala, India

## Abstract

We report here the whole-genome sequence of six clinical isolates of influenza A(H1N1)pdm09, isolated from Kerala, India. Amino acid analysis of all gene segments from the A(H1N1)pdm09 isolates obtained in 2014 and 2015 identified several new mutations compared to the 2009 A(H1N1) pandemic strain.

## GENOME ANNOUNCEMENT

In India, starting in the winter of 2014 to 2015, health officials observed a significant increase in the number of H1N1 case fatalities compared to the pandemic period of 2009 to 2010 (1). Influenza A virus constantly undergoes antigenic evolution and it is important to continually examine the evolutionary changes in the circulating strains each year. Whole-genome sequencing provides the necessary evidence for understanding the origins and genetic variations of viruses, for proper implementation of public health management, including vaccine development (2).

Here, we report the whole-genome sequence of six clinical isolates of influenza A(H1N1)pdm09, isolated from Kerala, a state in south India. The study was approved by the Institutional Human Ethics Committee of the Rajiv Gandhi Centre for Biotechnology (no. IHEC/1/2013/01). Isolation was carried directly from nasopharyngeal swabs collected from patients with a clinical diagnosis of influenza-like illness and confirmed by real-time PCR. Six isolates were selected for whole-genome sequencing (one isolate from 2009, two isolates from 2014, and three isolates from 2015). All eight influenza gene segments were amplified by reverse transcription-PCR using the WHO–CDC (Atlanta) recommended whole-genome primers (3). Each segment was amplified in four to six fragments of 400 to 600 bp with 100-bp overlap in order to get four-fold sequence coverage. Sequencing was carried out using the BigDye terminator version 3.1 cycle sequencing kit (ABI, Foster City, CA, USA) and processed for capillary electrophoresis on an ABI 3500 DNA analyzer. Data analysis was done with Sequence Scanner version 2 software, and Bio Edit version 7.2.5 was used for sequence alignments.

All gene segments were aligned against the California strain of 2009 (A/California/04/2009), as well as the globally circulating strains. Compared to the pandemic period, a large number of amino acid substitutions were observed for D97N, N129D, K163Q, V173I, S185T, A256T, K283E, I295V, E491G, and E499K in the A(H1N1)pdm09 isolates from 2014 to 2015. The two variations, K163Q and S185T, were observed in the Sa and Sb domains, respectively. Phylogenetic analysis of the HA sequences of these isolates showed that they belonged to clade 6B (4). None of the isolates were resistant to neuraminidase inhibitor drugs, but the neuraminidase gene showed mutations for N44S, V241I, and N-369K, which have been reported to facilitate stability in the virus. Similarly, the majority of amino acid substitutions observed during the 2009 pandemic period—namely, those for PB2-I354L, PB1-I397M, I435T, PA-P224S, NS-I123V, and NP-V100I—persisted in the isolates from 2014 to 2015, with the accumulation of several new mutations in PB2-R54K, M66I, D195N, R293K, V344M, R368R, W537L, V731I, S453T, R299K, T303S, PB1-V113I, G154D, PA-I621V, G684R, R266H, S522G, R559K, L649I, V100I, N321K, I330V, K361R, R362K, Ns-G51S, and NP- A22T.

### Accession number(s).

The whole-genome sequences obtained from six Kerala, South Indian A(H1N1)pdm09 clinical isolates from 2009, 2014, and 2015 have been deposited in GenBank under the accession numbers listed in Table 1.

**TABLE 1:** Accession numbers of whole-genome sequences of A(H1N1)pdm09 clinical isolates

	GenBank accession number by gene^a^							
Isolate	PB2	PB1	PA	HA	M	NP	NA	NS
RGCB140815	KU922529	KU922535	KU922541	KY888150	KU922555	KU922548	KY888153	KU922561
RGCB141437	KU922530	KU922534	KU922540	KY888151	KU922554	KU922547	KY888154	KU922560
RGCB145385	KU922531	KU922536	KU922542	KY888149	KU922556	KU922549	KU922552	KU922562
RGCB145751	KY888152	KU922538	KU922544	KY888148	KU922558	KU922551	KY888155	KU922564
RGCB145808	KU922532	KU922537	KU922543	KU922545	KU922557	KU922550	KY888156	KU922563
RGCBA923	KY888158	KU922533	KU922539	KM203128	KU922553	KU922546	KY888157	KU922559

a PB2, polymerase basic 2; PB1, polymerase basic 1; PA, polymerase acidic; HA, hemagglutinin; M, matrix; NP, nucleoprotein; NA, neuraminidase; NS, nonstructural.
